# How Have Public Safety Personnel Seeking Digital Mental Healthcare Been Affected by the COVID-19 Pandemic? An Exploratory Mixed Methods Study

**DOI:** 10.3390/ijerph17249319

**Published:** 2020-12-13

**Authors:** Hugh McCall, Janine Beahm, Caeleigh Landry, Ziyin Huang, R. Nicholas Carleton, Heather Hadjistavropoulos

**Affiliations:** 1 Department of Psychology, University of Regina, 3737 Wascana Pkwy, Regina, SK S4S 0A2, Canada; Hugh.McCall@uregina.ca (H.M.); Janine.Beahm@uregina.ca (J.B.); Caeleigh.Landry@uregina.ca (C.L.); Nick.Carleton@uregina.ca (R.N.C.); 2 PSPNET, University of Regina, 2 Research Drive, Regina, SK S4T 2P7, Canada; Ziyin.Huang@uregina.ca

**Keywords:** public safety personnel, first responders, COVID-19, coronavirus, pandemic, mental health, internet-delivered cognitive behavioral therapy

## Abstract

Public safety personnel (PSP) experience unique occupational stressors and suffer from high rates of mental health problems. The COVID-19 pandemic has impacted virtually all aspects of human life around the world and has introduced additional occupational stressors for PSP. The objective of this study was to explore how PSP, especially those seeking digital mental health services, have been affected by the pandemic. Our research unit, PSPNET, provides internet-delivered cognitive behavioral therapy to PSP in the Canadian province of Saskatchewan. When the pandemic spread to Saskatchewan, PSPNET began inquiring about the impact of the pandemic on prospective clients during the eligibility screening process. We used content analysis to analyze data from telephone screening interviews (*n* = 56) and descriptive statistics to analyze data from a questionnaire concerning the impacts of COVID-19 (*n* = 41). The results showed that most PSP reported facing several novel emotional challenges (e.g., social isolation, boredom, anger, and fear) and logistical challenges (e.g., related to childcare, finances, work, and access to mental healthcare). Most participants indicated they felt at least somewhat afraid of contracting COVID-19 but felt more afraid of their families contracting the virus than themselves. However, few participants reported severe challenges of any kind, and many (40%) indicated that they had not been significantly negatively impacted by the pandemic. Overall, the results suggest that PSP are not expressing significant concern at this time in meeting the novel challenges posed by COVID-19. Continued research will be required to monitor how diverse PSP populations and treatment outcomes are affected by the pandemic as the situation evolves.

## 1. Introduction

Public safety personnel (PSP) include border services officers, public safety communications officials, correctional workers, firefighters (career and volunteer), Indigenous emergency managers, operational intelligence personnel, paramedics, police (municipal, provincial, and federal), and search and rescue personnel [1]. Past research has found that PSP report much higher levels of mental health problems (i.e., 44.5% screen positive for one or more mental health disorders) [2] and suicidal behaviors (i.e., ideation, planning, and attempts) [3] than are found in the general population [4]. The most common mental disorders among PSP appear to be major depressive disorder (26.4%) and post-traumatic stress disorder (PTSD; 23.2%) [2]. PSP also report higher rates of exposures to potentially psychologically traumatic events [1] than the general population [5,6] as a result of their vocations, which may contribute to the higher reports of mental health challenges [7]; however, there is also evidence that PSP workplace environments [8] and attitudes towards mental health [9,10] exacerbate mental health challenges.

The COVID-19 pandemic has spread worldwide since December 2019, leading to unprecedented changes in daily life (e.g., stay-at-home orders, business closures, and mask mandates) [11]. The changes have contributed to widespread fear and uncertainty regarding the pandemic [12]. Research from previous pandemics suggests that the psychological impact of the COVID-19 pandemic will be greater than the physical impact of the virus [13,14]. As such, an increasing body of research is focusing on the impact of the COVID-19 pandemic on the general population [15,16,17,18]. More than 25% of the population in China are struggling with moderate to severe anxiety or stress as a result of the COVID-19 pandemic [16,19]. Among Australians seeking digital mental healthcare, Titov and colleagues found that many presented with a range of concerns related to the COVID-19 pandemic, including job loss, financial concerns, risk of infection, and self-isolation [18]. Taylor and colleagues found that approximately 16% of a representative US and Canadian population sample appear to be suffering from severe COVID Stress Syndrome symptoms, referring to concerns in diverse areas such as traumatic stress symptoms, fears of contamination, and xenophobia [20]. However, very few studies have been conducted on the impact of COVID-19 on PSP, who are likely to be uniquely affected (e.g., through the introduction of novel occupational stressors) [21]. Research that has been conducted suggests that PSP and frontline healthcare workers are being disproportionately impacted by the COVID-19 pandemic as a function of their vocational duties [22].

Of concern, PSP face many barriers to accessing mental healthcare, including concerns about stigma, time constraints, and geographic and financial barriers [23,24]. Internet-delivered cognitive behavioral therapy (ICBT) is private, accessible at virtually any time and location, and cost-effective [25,26], which can help PSP overcome most barriers. Indeed, research shows that PSP report favorable attitudes towards ICBT [23,27]. Our research unit, PSPNET, launched a free-to-access ICBT program for PSP in the Canadian province of Saskatchewan in January 2020, which was shortly before the COVID-19 pandemic reached Saskatchewan. The temporality of the launch has provided us with a serendipitous opportunity to explore how treatment-seeking PSP have been impacted by COVID-19.

Our objective was to explore the impact of the COVID-19 pandemic on PSP seeking ICBT. The project was focused on the diversity, frequency, and intensity of concerns PSP have regarding COVID-19. We expected PSP would report concerns about novel stressors in various life domains (e.g., work, home, and social life). However, this research was exploratory in scope, and we did not make any specific hypotheses.

## 2. Materials and Methods

### 2.1. Context and Timeline

PSPNET began delivering its first ICBT program, The PSP Wellbeing Course, in Saskatchewan on 5 December 2019. It officially launched the program across the province on 29 January 2020, just two days after the first case of COVID-19 was confirmed in Canada [28]. The first presumptive case of COVID-19 in Saskatchewan was confirmed on 12 March 2020 [29], and a provincial state of emergency was declared on 18th March [30]. Over the following weeks, the province of Saskatchewan released several public health orders implementing a variety of restrictions (e.g., suspending in-person classes at primary and secondary schools and restricting access to restaurants and recreational facilities) [31].

### 2.2. Procedure and Participants

**Figure 1:**
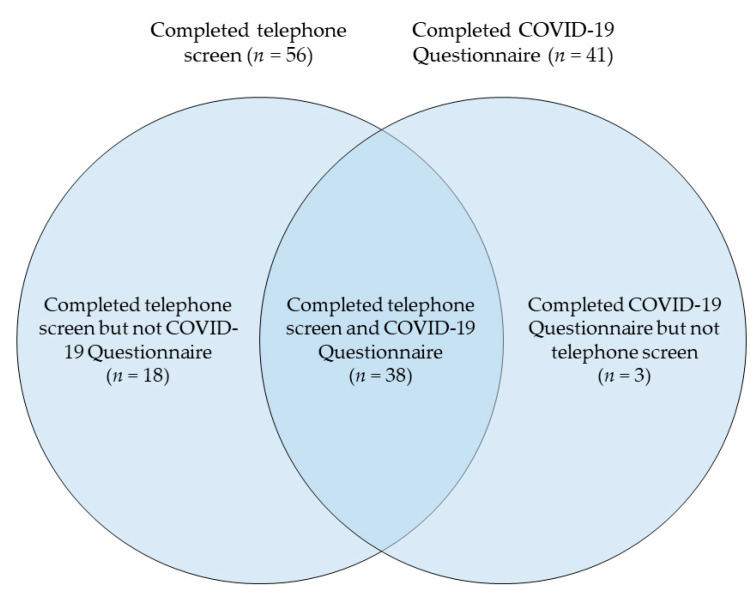
Number of participants who completed telephone screen and COVID-19 Questionnaire.

Before enrolling in PSPNET’s ICBT programs, PSP must complete a brief online screening questionnaire; then a more thorough battery of online screening questionnaires; and finally, a screening interview with a PSPNET therapist by phone. When the COVID-19 pandemic spread to Saskatchewan, PSPNET therapists began asking prospective clients, during telephone screens, how they had been impacted by the COVID-19 pandemic. On 13 April 2020, PSPNET added a questionnaire concerning the impacts of COVID-19 on PSP’s lives to its battery of online screening questionnaires. All Saskatchewan PSP who completed the COVID-19 Questionnaire and/or the telephone screening between 18 March and 11 September 2020 were included in this study. To be eligible to participate in this study, each participant first had to complete the brief online screening questionnaire and report (a) being a Saskatchewan resident, (b) being at least 18 years old, (c) having internet access, (d) having one to two hours per week for eight weeks to engage in ICBT, and (e) being willing to provide a medical contact. A total of 59 participants were included in the present study, 41 of whom completed the COVID-19 Questionnaire and 56 of whom completed the telephone screen. Of note, three participants completed the COVID-19 Questionnaire but could not be reached for a telephone screen, and 18 completed the telephone screen before we began administering the COVID-19 Questionnaire. The overlap between the subsets of participants who completed the telephone screen and the COVID-19 Questionnaire is displayed below in Figure 1. All participants consented to the use of their data for research purposes during the online screening.

### 2.3. Data and Analysis

We employed a mixed methods approach. Specifically, we used a qualitative content analysis approach to analyze telephone screening and descriptive statistics to analyze COVID-19 Questionnaire data. We conducted the qualitative and quantitative analyses concurrently and triangulated the results to cross-validate our findings and help ensure their completeness without prioritizing either approach [32]. These analyses are described in detail below.

During the online screening, we administered (1) a demographics questionnaire; (2) the 9-item Patient Health Questionnaire (PHQ-9) [33]; (3) the 7-item Generalized Anxiety Disorder questionnaire (GAD-7) [34]; (4) the PTSD Checklist for DSM-5 (PCL-5) [35]; and (5) an 18-item questionnaire concerning fears of COVID-19 and impacts of COVID-19 in various life domains, which was adapted from the Fear of Illness and Virus Evaluation [36]. The PHQ-9, GAD-7, and PCL-5 have all shown good psychometric properties [33,34,37,38]. Scores on the PHQ-9 range from 0 to 27, with a score of 10 or greater indicating a likely diagnosis of major depressive disorder [39]; however, a cut-off score of 14 has recently been recommended [40]. GAD-7 scores range from 0 to 21, and a score of 10 or greater indicates a likely diagnosis of generalized anxiety disorder [34,37]. On the PCL-5, scores range from 0 to 80, and among several proposed cut-off scores, at least two research groups have recommended a score of 33 as indicative of likely PTSD [38,41]. Participants’ quantitative responses to these questionnaires were assessed using descriptive statistics.

During the follow-up telephone screen, PSPNET therapists recorded summaries of participants’ symptoms and contributing factors in the “Notes” section on the therapists’ website portal. Author J.B. exported these notes from the website, removed identifying information, and imported them into NVivo 12 [42] for qualitative analyses. She conducted a directed content analysis [43] of the notes and coded the data by meaning units. A meaning unit was defined as any mention of a negative impact related to COVID-19. She created the initial coding strategy using the COVID-19 Questionnaire as a guiding framework. The initial codes were then grouped into two larger domains (negative emotions and logistical impacts). Following the initial coding, authors J.B., H.H., C.L., and H.M. met as a group and reviewed the domains and the codes, including each individually coded piece of data, within each domain. We adjusted code names and the placement of individually coded data following discussion. We saved a copy of the previous coding strategy and notes on changes made to establish an audit trail. The number of clients who endorsed each theme are reported to emphasize commonly endorsed themes, but these numbers are not meant to suggest generalizability to a wider population.

## 3. Results

### 3.1. Participant Characteristics

Most participants self-identified as White (*n* = 46, 78%), women (*n* = 34, 58%), married or common law (*n* = 32, 54%), without children (*n* = 32, 54%), and living in communities with a population of less than 100,000 (*n* = 32, 54%). Participants reported working in a range of PSP sectors. On average, participants were 39.73 years old (*SD* = 10.20) and reported symptoms around the clinical cut-offs on measures of depression (mean PHQ-9 score = 12.17, *SD* = 6.28), anxiety (mean GAD-7 score = 10.63, *SD* = 5.77), and PTSD (mean PCL-5 score = 30.76, *SD* = 19.61). The demographic and clinical characteristics of our sample are displayed in Table 1.

### 3.2. Results of COVID-19 Questionnaire

Results of the nine COVID-19 Questionnaire items concerning fear of COVID-19 are presented in detail in Table 2. For each of these items, most participants responded, “I am not afraid of this at all”, or “I am afraid of this some of the time”, with relatively few participants reporting more frequent fears. On average, participants reported the greatest fear about causing others to contract COVID-19 and family members contracting COVID-19. They reported the least fear about going to the hospital because of COVID-19, dying from COVID-19, and pets contracting COVID-19. Most participants reported feeling afraid of contracting COVID-19 themselves some of the time (*n* = 22, 54%) or not at all (*n* = 16, 39%).

Results of the nine COVID-19 Questionnaire items concerning the impacts of COVID-19 are presented in Table 3. Only one participant (2%) had contracted COVID-19, and only five (12%) were close to others who had contracted COVID-19. Most (*n* = 29, 71%) reported experiencing a moderate financial impact, but very few (*n* = 4, 10%) reported having lost their job or income due to COVID-19. Most participants (*n* = 23, 56%) expressed concern about their ability to maintain physical distance from others at work. Few reported issues with childcare (*n* = 6, 32% of participants with children). Emotionally, most participants reported no difficulties with feeling socially isolated due to COVID-19 (*n* = 26, 63%) and indicated that fear of COVID-19 had not interfered with their enjoyment of life (*n* = 24, 59%). A sizeable minority (*n* = 18, 44%) reported that COVID-19 had not caused them strong emotions.

### 3.3. Results of Qualitative Analyses of Telephone Screens

A majority of participants who completed telephone screens (*n* = 33/56, 59%) reported that the COVID-19 pandemic was impacting them to some extent in a negative way. Among these participants, concerns about the COVID-19 pandemic fell under two broad domains, including: negative emotions (*n* = 22/56, 39%) and logistical complications (*n* = 17/56, 30%). Participants who were experiencing negative emotions reported they were experiencing increased negative emotions due to a range of factors. Some participants reported concerns about others, such as family members, contracting COVID-19, becoming sick, and possibly dying (*n* = 12/56, 21%), and slightly fewer reported having concerns about contracting the virus themselves, becoming sick, and possibly dying (*n* = 8/56, 14%). They also reported negative emotions due to isolation (*n* = 5/56, 9%) and boredom due to business closures impeding life enjoyment (*n* = 3/56, 5%). Some participants reported that the COVID-19 pandemic is causing them to experience increased stress or negative emotions but did not elaborate further (*n* = 5/56, 9%). Responses under the logistical implications domain included participants’ reports of negative impacts on their work life (*n* = 6/56, 11%), such as concerns about increased call volumes, the inability of the healthcare system to manage the impact of COVID-19, and concerns about management not taking the pandemic seriously and not providing frontline workers adequate personal protective equipment. Some participants also reported financial concerns (*n* = 6/56, 11%) due to closures and physical distancing measures causing a loss of finances from secondary businesses, the participants’ partners losing their jobs/incomes, and reduced shift loads. Another logistical concern identified by participants was how closures were reducing their own or a family member’s ability to access support services, particularly mental health services (*n* = 5/56, 9%). A few participants noted the pandemic is causing increased stress due to changes in home life (*n* = 4/56, 7%) (e.g., not having access to childcare and having to navigate a new routine). Two participants stated that they reached out to PSPNET because of reduced access to other mental health services. See Table 4 for a summary of these results.

## 4. Discussion

This study used a mixed methods approach to explore how the COVID-19 pandemic has impacted a diverse sample of PSP seeking ICBT in Saskatchewan. The qualitative and quantitative analyses produced similar results. Most participants reported that the COVID-19 pandemic had negatively impacted their lives emotionally and logistically, but few participants reported severe impacts, and over 40% of clients who completed the telephone screen did not report any negative impacts of COVID-19. Emotional impacts included feelings of isolation, boredom, anger, and fear. Participants reported clinically significant symptoms of depression, anxiety, and PTSD, but the extent to which these symptoms were related to the COVID-19 pandemic was unclear. Logistical impacts included difficulties with childcare, financial difficulties, novel work-related stressors, and difficulty accessing mental health support. Although less than a third of participants who had children reported that the pandemic has caused childcare issues, it is likely that some participants’ children were adolescents or adults and did not require childcare.

These findings indicate that many PSP are confronted with a range of novel stressors resulting from the COVID-19 pandemic. They also suggest that PSP are generally expressing less distress or concern about these challenges than we had expected, especially in light of the fact that PSP were reporting clinically significant symptoms of depression, anxiety, and PTSD. The results have helped our therapists understand how PSP are experiencing the pandemic. This information may be informative for other programs designed to help PSP cope with these stressors so they can maintain the best possible mental health and focus on their important roles in keeping our communities safe.

Overall, our findings align with those of other studies. The current results are consistent with anecdotal results reported through the Canadian Institute for Public Safety Research and Treatment COVID-19 Resource Readiness Project [22], which suggested that the pandemic has presented Canadian PSP with various novel stressors and emotional and logistical challenges (e.g., risk of infection and self-isolation). However, the present study is the first study that we are aware of to have systematically explored the impacts of the pandemic on PSP. Consistent with prior research in non-PSP populations, many participants reported fearing that they [15,18,20,44] or their families [16,18] may become infected with COVID-19. Furthermore, like the general population, participants reported financial consequences of the pandemic and difficulty with social isolation [15,18,20]. However, participants reported facing unique occupational challenges that are unlikely to be faced by many in the general population (e.g., insufficient personal protective equipment and increased call volume). Finally, other studies have identified several issues regarding the COVID-19 pandemic that we did not assess, including fears of socio-economic consequences, xenophobia, reassurance seeking and compulsive checking, and traumatic stress symptoms [17].

This study has several important limitations. First, our sample was small. Second, we were unable to include all participants in both our qualitative and quantitative analyses. Third, our results may not generalize to other provinces or countries, particularly those that had been impacted more severely by the pandemic than Saskatchewan at the time that this study was conducted. Relatedly, results may not generalize to non-treatment-seeking PSP populations. Fourth, we were unable to address changes over time in how the pandemic impacted PSP. Nevertheless, this study provides initial evidence concerning how PSP have been impacted by COVID-19 and sets the stage for ongoing monitoring of the effect of the pandemic on PSP.

Future research can expand upon these findings in several ways. First, PSPNET will examine the outcomes of PSPNET programs, including the extent to which PSP are using them to manage stressors related to COVID-19. Second, further research will be required to evaluate how other PSP populations have been impacted by the pandemic. Due to the dynamic nature of the COVID-19 pandemic, researchers will need to be ready to respond by asking and addressing new research questions as they emerge and exploring changes in the impacts of COVID-19 over time. In particular, it is possible that PSP in Saskatchewan have been more affected by COVID-19 throughout the fall of 2020 due to increases in active case numbers from the time this research was carried out [31].

## 5. Conclusions

The present findings suggest that PSP seeking digital mental health treatment in Saskatchewan have been confronted with many of the same emotional and logistical challenges as the general population, in addition to several unique occupational stressors. Participants reported experiencing social isolation, boredom, anger, and fear that they and, in particular, their families might contract COVID-19. They reported that the pandemic has introduced new challenges related to childcare, finances, work, and access to mental healthcare. However, few participants described these impacts as severe, and many indicated that the pandemic had not significantly affected them. The present study is the first study, that we are aware of, to systematically elucidate the impacts of the COVID-19 pandemic on PSP and can inform policies and programs designed to help PSP cope. Further research will be required to monitor how diverse PSP populations have been affected and continue to be affected by the COVID-19 pandemic as the situation unfolds.

## Figures and Tables

**Table 1:** Participant characteristics.

Characteristic		Participants Who Completed COVID-19 Questionnaire (*n* = 41)	Participants Who Completed Telephone Screen (*n* = 56)	All Participants (*N* = 59)
Age, *M* (*SD*)		40.13 (10.80)	39.73 (10.20)	39.73 (10.20)
Gender, *n* (%)				
	Woman	24 (59)	32 (57)	34 (58)
	Man	16 (39)	24 (43)	24 (41)
	Other	1 (2)	0	1 (2)
Ethnicity, *n* (%)				
	White	32 (78)	45 (80)	46 (78)
	First Nations, Inuit, or Metis	7 (17)	7 (13)	9 (15)
	Other	2 (5)	4 (7)	4 (7)
Married or common law, *n* (%)				
	Yes	21 (51)	31 (55)	32 (54)
	No	20 (49)	25 (45)	27 (46)
Children, *n* (%)				
	Yes	19 (46)	27 (48)	27 (46)
	No	22 (54)	29 (52)	32 (54)
Community size, *n* (%)				
	Population of 100,000 or greater	15 (37)	30 (54)	27 (46)
	Population under 100,000	26 (63)	26 (46)	32 (54)
PSP Sector, *n* (%)				
	Police	14 (34)	21 (38)	21 (36)
	Corrections	8 (20)	9 (16)	10 (17)
	Emergency Medical Service	8 (20)	11 (20)	12 (20)
	Fire	4 (10)	4 (7)	4 (7)
	Dispatch/Communications	3 (7)	5 (9)	5 (8)
	Other	4 (10)	6 (11)	7 (12)
Clinical characteristics, *M* (*SD*)				
	PHQ-9 score	12.76 (6.30)	12.11 (6.16)	12.17 (6.28)
	GAD-7 score	11.51 (5.92)	10.79 (5.74)	10.63 (5.77)
	PCL-5 score	31.95 (19.68)	30.93 (20.11)	30.76 (19.61)

**Table 2:** Results of COVID-19 Questionnaire items concerning fear of COVID-19.

Item	Frequency of Responses, *n* (%)				Mean Response, *SD*
	I Am Not Afraid of This at All (0)	I Am Afraid of This Some of the Time (1)	I Am Afraid of This Most of the Time (2)	I Am Afraid of This All of the Time (3)	
1. I am afraid I may get COVID-19.	16 (39)	22 (54)	2 (5)	1 (2)	0.71 (0.68)
2. I am afraid I will get very, very sick if I catch COVID-19.	19 (46)	18 (44)	1 (2)	3 (7)	0.71 (0.84)
3. I am afraid I will have to go to the hospital because of COVID-19.	26 (63)	14 (34)	0	1 (2)	0.41 (0.63)
4. I am afraid I might die if I get COVID-19.	27 (66)	12 (29)	1 (2)	1 (2)	0.41 (0.67)
5. I am afraid my pet might get COVID-19.	33 (80)	6 (15)	2 (5)	0	0.24 (0.54)
6. I am afraid a family member might get sick or die because of COVID-19.	5 (12)	21 (51)	9 (22)	6 (15)	1.39 (0.89)
7. I am afraid I may do something that would cause someone else to get COVID-19.	12 (29)	20 (49)	4 (10)	5 (12)	1.05 (0.95)
8. I am afraid a friend might get sick or die because of COVID-19.	18 (44)	18 (44)	5 (12)	0	0.68 (0.69)
9. I am afraid people in the world might get sick or die because of COVID-19.	25 (61)	12 (29)	4 (10)	0	0.49 (0.68)

Note. The data in this table were obtained from 41 of our 59 participants; the other 18 participants completed the online prescreen before we added the COVID-19 Questionnaire to it.

**Table 3:** Results of COVID-19 Questionnaire items concerning impacts of COVID-19.

Impact		Participants (*n* = 41)
COVID-19 status, *n* (%)		
	Recovered	1 (2)
	Never infected	40 (98)
Someone close to participant has had COVID-19, *n* (%)		
	Yes	5 (12)
	No	36 (88)
Childcare issues related to COVID-19, *n* (%)		
	Yes	6 (15)
	No	13 (32)
	No children	22 (54)
Lost job or income due to COVID-19, *n* (%)		
	Yes	4 (10)
	No	37 (90)
Impact of COVID-19 on ability to meet financial obligations, *n* (%)		
	Too soon to tell	1 (2)
	No impact	4 (10)
	Minor impact	4 (10)
	Moderate impact	29 (71)
	Major impact	3 (7)
Concern about ability to maintain physical distance at work, *n* (%)		
	Yes	23 (56)
	No	18 (44)
Difficulties with feeling socially isolated due to COVID-19, *n* (%)		
	Yes	15 (37)
	No	26 (63)
Being afraid of COVID-19 has gotten in the way of enjoying life, *n* (%)		
	Not true for participant at all	24 (59)
	Somewhat true	16 (39)
	Mostly true	1 (2)
	Definitely true	0
Being afraid of COVID-19 has caused strong emotions, *n* (%)		
	Not true for participant at all	18 (44)
	Somewhat true	17 (41)
	Mostly true	4 (10)
	Definitely true	2 (5)

Note. The data in this table were obtained from 41 of our 59 participants; the other 18 participants completed the online prescreen before we added the COVID-19 Questionnaire to it.

**Table 4:** Results of qualitative analyses of telephone screens concerning impacts of COVID-19.

Domain/Theme		Definition	Participants (*n* = 56)
Negative emotions, *n* (*%*)			22 (39)
	Fear of others contracting COVID-19	Fears about loved ones or co-workers contracting the virus, getting sick, or dying.	12 (21)
	Fear of contracting COVID-19	Fears about themselves contracting the virus, getting sick, or dying.	8 (14)
	Isolation	Negative emotions resulting from physical distancing measures leading to isolation.	5 (9)
	Boredom	Boredom due to closures.	3 (5)
	General stress	Increased overall stress levels.	5 (9)
Logistical impacts, *n* (%)			17 (30)
	Impact on work	Concerns included increased call volumes, the inability of the healthcare system to manage the impact of COVID-19, and concerns about management not taking the pandemic seriously.	6 (11)
	Financial concerns	Financial concerns included reduced business in secondary businesses, partners losing job/income, or reduced shifts/hours.	6 (11)
	Reduced access to support	Closures affecting access to support for participants themselves or their family members.	5 (9)
	Impact on home life	Concerns were related to not having access to childcare and having to navigate a new routine.	4 (7)

Note. The data in this table were obtained from 56 of our 59 participants; 3 participants did not complete a telephone screen.
